# Vitamin-D Receptor-Gene Polymorphisms Affect Quality of Life in Patients with Autoimmune Liver Diseases

**DOI:** 10.3390/nu12082244

**Published:** 2020-07-27

**Authors:** Agnieszka Kempinska-Podhorodecka, Monika Adamowicz, Mateusz Chmielarz, Maciej K. Janik, Piotr Milkiewicz, Malgorzata Milkiewicz

**Affiliations:** 1 Department of Medical Biology, Pomeranian Medical University, 70-111 Szczecin, Poland; monikadamowicz@gmail.com (M.A.); ch.mateusz94@gmail.com (M.C.); milkiewm@pum.edu.pl (M.M.); 2 Liver and Internal Medicine Unit, Medical University of Warsaw, 02-097 Warsaw, Poland; mjanik24@gmail.com (M.K.J.); p.milkiewicz@wp.pl (P.M.); 3 Translational Medicine Group, Pomeranian Medical University, 70-111 Szczecin, Poland

**Keywords:** autoimmune hepatitis, primary biliary cholangitis, vitamin D, health-related quality of life, mental well-being

## Abstract

Vitamin D deficiency has been associated with depressive symptoms and reduced physical functioning. The aim of the study was to characterize the relationship between polymorphisms of the vitamin D receptor (VDR) gene and the quality of life in patients with autoimmune hepatitis (AIH) and primary biliary cholangitis (PBC). Three polymorphisms of the *VDR* gene (*TaqI-rs731236*, *BsmI-rs1544410*, and *ApaI-rs7975232*) were analyzed in patients with AIH (*n* = 142) and PBC (*n* = 230) and in healthy individuals (*n* = 376). Patient quality of life was assessed by validated questionnaires such as Medical Outcomes Study Short-Form 36 (SF-36), State Trait Anxiety Inventory (STAI), Modified Fatigue-Impact Scale (MFIS), Patient-Health Questionnaire 9 (PHQ-9), and PBC-40. The *TaqI* C and *ApaI* A alleles are risk alleles in both AIH and PBC, and a significant dominance of the A allele in *BsmI* was observed in AIH patients. In terms of quality of life, the presence of the CC or CT *TaqI* genotype was associated with emotional reactions, including the fatigue and the cognitive skills of patients with PBC, whereas in the group of AIH patients, homozygotes CC of *TaqI*, AA of *BsmI*, and AA of *ApaI* had worse physical, social, emotional, and mental function. The genetic variations of *VDR* gene can influence individual susceptibility to develop chronic autoimmune liver diseases such as AIH and PBC and affect quality of life.

## 1. Introduction

Autoimmune liver diseases, such as primary biliary cholangitis (PBC) and autoimmune hepatitis (AIH), have complex etiologies and are characterized by the progressive destruction of liver structures through autoimmunity mechanisms [1,2]. The vast majority of patients with PBC (80%–90%) are women [3]. The reaction between antimitochondrial antibodies (AMA) and pyruvate dehydrogenase complex-E2 (PDC-E2), located in the inner mitochondrial membrane, underlies the pathogenesis of PBC [4]. The most common clinical symptoms are persistent pruritus and chronic fatigue; however, a substantial percentage of patients may experience no symptoms of liver disease [1]. In biochemistry, elevated alkaline phosphatase and gamma-glutamyltranspeptidase activity, hypercholesterolemia, and often an increase in IgM level are observed. Histologically, the disease is characterized by bile-duct damage leading to chronic cholestasis, progressive fibrosis, and liver cirrhosis [3].

AIH is a disease that affects women more often than men regardless of age or ethnicity [5]. Biochemically, it is characterized by elevated transaminases and hypergammaglobulinemia. It can be divided into two types depending on autoantibodies; Type 1 is the most common and confirmed by the presence of an antinuclear antibody (ANA) or anti-smooth muscle antibody (ASMA). Histologically, AIH is marked by interface hepatitis, emperipolesis, and hepatocyte rosettes [6].

A substantial impairment of health-related quality of life (HRQoL) was reported in both PBC and AIH, with chronic fatigue and depression occurring in a significant proportion of patients with both diseases and pruritus affecting patients with PBC [7,8].

Vitamin D3 inhibits parathyroid-hormone secretion, cell proliferation, and adaptive immunity [9]. The activity of autoimmune diseases is influenced by vitamin-D3 deficiency [10], and this nonclassical effect of vitamin D is associated with the presence of the vitamin-D receptor (VDR) on numerous cells in the immune system. It was demonstrated that vitamin D has an impact on the Th1 lymphocytes responsible for the production of interleukin 2, tumor necrosis factor-alpha (TNA-α), and interferon gamma (IFNγ) [11]. The secretion of these cytokines can inhibit calcitriol (1.25(OH)_2_D_3_—the active metabolite of vitamin D), which translates into the alleviation of the inflammatory reaction. This substance also has a promoting effect on T regulatory cells that are also responsible for turning off the immune-system response, which may be crucial in the treatment of autoimmune diseases associated with excessive responses by the immune system [12]. Furthermore, the presence of VDR was observed on Th2 cells that, after activation, produce interleukins 4 and 10 [13]. Vitamin D enhances the diversification of macrophages and their bactericidal effect; it also inhibits the maturation of dendritic cells, which is essential in autoimmune diseases [14]. Consequently, attempts were made to use vitamin D_3_ as a biopreparate capable of treating humans through the immunomodulation of the immune system [15].

Recent studies showed that vitamin D is also involved in neurotransmission and neuroprotection, and its receptor (VDR) is present in brain tissue, like glial cells, hippocampus, thalamus, or neurons [16]. In turn, the polymorphism of the *VDR* gene modulates *VDR* expression that can affect the vitamin D_3_ signaling cascade [17,18]. A combination of these factors may suggest the impact of polymorphisms of the *VDR* gene on cognitive dysfunction, thus reducing the quality of life of patients with PBC and AIH.

Therefore, bearing in mind the results of our own study regarding the reduced expression of *VDR* in patients with PBC [19], the clinical symptoms of patients with PBC and AIH (including chronic fatigue and insomnia) [20,21], and reports on the protective effect of vitamin D on vessels and nerves, the aims of this study were characterizing the relationship between *VDR* gene polymorphisms (*BsmI and ApaI*, located in an intron between exons 8 and 9, and *TaqI* C > T located in exon 9) and the HRQoL in patients with a clinical diagnosis of PBC and AIH using validated scale tools and clinical-data forms.

## 2. Materials and Methods

### 2.1. Patients

Two-hundred-and-thirty patients with PBC (213 females and 17 males; median age at diagnosis 55; range 28–90 years) and 142 patients with AIH (111 females and 31 males; median age at diagnosis 32; range 24–64 years) were include into this study. Vitamin D supplementation was recommended in all patients with PBC. Main laboratory and demographic data of the included patients are presented at Table 1.

All patients with PBC met The European Association for the Study of the Liver (EASL) criteria for the diagnosis of PBC [22]. One-hundred-and-fifty-eight (68.7%) patients who had histological/clinical/imaging features consistent with liver cirrhosis AIH were diagnosed according EASL’s Clinical Practice Guideline [2] for this condition and sixty-five (45.8%) of them had features of liver cirrhosis in histology.

A control group of 376 blood donors from the Regional Blood Donor Center in Szczecin (Poland), (344 females and 32 males; median age at enrollment 28; range 18–66 years) was investigated. All participants had a medical check-up. A good state of health was a prerequisite to qualify for blood donation. Each participant provided their written informed consent. All materials were deposited in the Department of Medical Biology, Pomeranian Medical University in Szczecin.

The study was approved by the Bioethical Committee of the Pomeranian Medical University in Szczecin, 2011, no. KB-0012/57/11.

### 2.2. VDR Genotyping

DNA was extracted from peripheral blood mononuclear cells using the DNeasy Blood and Tissue Kit (Qiagen, Hilden, Germany). Genotyping of three variants of *VDR* gene polymorphism (*TaqI-rs731236*, *BsmI-rs1544410*, *ApaI-rs7975232*) was carried out using real-time polymerase chain reaction using TaqMan probes (Applied Biosystems, Foster City, CA, Country; assay ID: C_2404008_10, C_8716062_10, C_28977635_10, respectively). Fluorescence analysis was conducted with Allelic Discrimination 7500 software v.2.0.2.

### 2.3. Health-Related Quality-of-Life Tools

Medical Outcomes Study Short Form 36 version 1.0 (*SF-36v1*, license no. QM011392-QualityMetric CT133208/OP018661) is a standardized questionnaire that contains 36 questions in 8 domains related to physical health (Physical Functioning, Role-Physical, Bodily Pain, General Health) and psychological well-being (Vitality, Social Functioning, Role-Emotional, Mental Health), which can be calculated in addition to two summary parameters: Physical-Component Summary and Mental-Component Summary [23].

PBC-40 was developed in 2005 and focuses on PBC [24]. The questionnaire consists of 40 questions related to the various aspects of chronic cholestatic liver disease: the worsening of chronic fatigue syndrome; feeling of health; skin pruritus; and cognitive, emotional, and social functions.

The Polish version of the Modified Fatigue-Impact Scale (MFIS) was used to assess the impact of fatigue on AIH patients’ life [25]. It is a modified form of the original Fatigue-Impact Scale. The questionnaire that included 21 items and a total MFIS score (range 0–84) is based on three subscales: physical (9 items, score range 0–36), cognitive (10 items, score range 0–40), and psychical (2 items, score range 0–8).

State Trait Anxiety Inventory (STAI) is a tool designed to measure the levels of state and trait anxiety [25]. The Polish version of this questionnaire was used in this study. This 40-item scale includes two subscales, state anxiety (1–20 items) and trait anxiety (20–40 items). Each item is given a weighted score of 1 to 4. Higher score suggests elevated levels of anxiety. Therefore, 0–20 results from both subscales represent no anxiety, a 41–60 score indicates midlevel anxiety, and results from 61 to 80 indicate severe anxiety.

Patient-Health Questionnaire 9 (PHQ-9) is a self-administered screening tool that is used to monitor the severity of depressive symptoms [26]. A questionnaire was validated for Polish population. PHQ-9 scores of 5–9, 10–14, 15–19, and 20–27 are the ranges for mild, moderate, moderately severe, and severe depression, respectively.

In patients with PBC, two questionnaires were used, SF-36 and PBC-40. In patients with AIH, SF-36, MFIS, PHQ-9, and STAI were applied.

### 2.4. Statistical Analysis

All statistical analyses were carried out using StatView version 5 software (SAS Institute Inc., Carry, NC, USA). The genotype and allelic frequencies were compared using a chi-squared test of association (Pearson). The odds ratio (OR) and 95% confidence interval (CI) for each variable were also estimated. Analysis of genotype frequency in regard to the clinical characteristics and HRQoL assessment of PBC and AIH patients was performed using ANOVA with Fisher’s protected least significant difference (PLSD). Data are shown as medians (and ranges) for demographic data, and as means and standard deviations (SD) for continuous variables of assessing HRQoL. *p*-values of less than 0.05 were considered to be statistically significant.

## 3. Results

The frequencies of all three *VDR* polymorphisms investigated in patients with PBC or AIH showed significant differences in comparison to the control group. The odds ratios (ORs) observed for the presence of these polymorphisms in the diseases and control groups are summarized in Table 2.

In PBC patients, the *TaqI* CC and CT genotypes were more prevalent in comparison to controls (36.5% vs. 11.7%, *p* < 0.001, and 51.3% vs. 42.6%, *p* = 0.04, respectively), whereas the TT genotype of *TaqI* was considerably less frequent than in the controls (12.2% vs. 45.7%, *p* < 0.001; Table 2). Similarly, in AIH patients, the *TaqI* CC genotype appeared more often than in the control group (22.5% vs. 11.7%, *p* = 0.002), while the TT was less frequent than in the controls (33.8% vs. 45.7%, *p* = 0.01; Table 2).

Regarding *BsmI* polymorphism, the frequency of the AA genotype was substantially higher in patients with AIH compared to controls (23.9% vs. 13.8%, *p* = 0.006; Table 2). The results of *ApaI* genotyping showed that the AA genotype occurred more frequently in both patients with PBC and AIH (27.4% vs. 19.7% in controls, *p* = 0.03, and 32.4% vs. 19.7% in controls; *p* = 0.002, respectively; Table 2).

Furthermore, analyses of frequencies of each allele in the three polymorphic sites clearly demonstrated that the *TaqI* C and *ApaI* A alleles were more prevalent in both PBC and AIH compared to in healthy individuals. Thus, the distribution of the *TaqI* C allele was 62.0% in PBC and 44.4% in AIH vs. 33.0% in controls (both *p* < 0.001); for the *ApaI* A allele, 51.4% in PBC and 54% in AIH vs. 45.7% in controls, *p* = 0.05 and *p* = 0.02, respectively. Additionally, 45.0% of AIH patients were carriers of the *BsmI* A allele in comparison to 36.8% controls, *p* = 0.02 (Table 3).

In addition, the clinical status and biochemical findings of the patients were examined in relation to *VDR* polymorphism. In PBC patients, *TaqI* and *BsmI* variants were associated with the histological features of cirrhosis regardless of cholestasis and autoimmune parameters in PBC. Thus, in patients with PBC who were cirrhotic at the diagnosis, 52.8% were *TaqI* CC, 39% were CT and 7.5% were TT genotype carriers (*p* < 0.0001 vs. *CC*). Similarly, in *BmsI* variant, 56.6% of cirrhotics were GG homozygous, 33.9% were GA heterozygous (*p* = 0.03 vs. *GG*), and 9.4% were *AA* homozygotes genotype (*p* < 0.0001 vs. *GG*). Other laboratory markers of the disease severity and enhanced level of AMAs, Gp210, and Sp100 antibodies failed to have any association to the analyzed polymorphisms. In contrast, in the group of AIH patients, the presence of the *VDR* polymorphisms did not correlate with the examined clinical and biochemical features (data not shown).

Regarding the quality of life of PBC patients, several domains of SF-36 and PBC-40 questionnaires were correlated only with the *TaqI* variant of *VDR* polymorphisms. The SF-36 general questionnaire demonstrated that PBC patients with CC and CT genotypes of *TaqI* variants had lower scores for Vitality (*p* = 0.01, and *p* = 0.04, respectively) and Role-Emotional (*p* = 0.03 and *p* = 0.04, respectively) than TT homozygotes did. The results of the PBC-40 questionnaires showed that carriers of CC and CT genotypes had significant cognitive impairment versus the TT genotype (*p* = 0.04 and *p* = 0.04, respectively). Furthermore, PBC patients with the *TaqI* CT genotype suffer from greater fatigue than patients with TT do (*p* = 0.04; Table 4).

In view of the fact that the *TaqI* C allele was associated with the increased risk of cirrhosis as well as reduced quality of life, we did an additional sub-group analysis corresponding to the presence of cirrhosis. We looked at features which came out significant, i.e., vitality and role emotional from SF-36 and cognitive function from PBC40. No significant differences between cirrhotic and non-cirrhotic patients were found, which may suggest that the allele itself, but not the presence of cirrhosis, exerts its negative effect on patients QoL.

Among patients with AIH, health-related quality of life was evaluated by the SF-36, STAI, MFIS, and PHQ-9 questionnaires. Differences between patients in relation to variants of *VDR* polymorphisms were observed only in SF-36 domains. The *TaqI* CC homozygotes scored fewer points for Role-Physical (*p* = 0.04) than the CT heterozygotes did, which, in turn, scored higher than TT homozygotes on Social-Functioning (*p* = 0.03; Table 5).

The *BsmI* AA homozygotes had lower scores for Physical-Component Summary than the GA heterozygotes did (*p* = 0.04; Table 6).

The most noticeable differences in quality of life measured by the generic SF-36 were observed in *ApaI* variants of *VDR* polymorphism. *ApaI* AA homozygotes had lower scores for 6 out of 10 factors, namely, Role-Physical (*p* = 0.02), Social Functioning (*p* = 0.04), Role-Emotional (*p* = 0.003), Mental Health (*p* = 0.04), Physical-Component Summary (*p* = 0.04), and Mental-Component Summary (*p* = 0.04) compared to CA heterozygotes (Table 7). Additionally, CA heterozygotes scored more points for Social Functioning (*p* = 0.01) than CC individuals did (Table 7).

## 4. Discussion

In this study, we analyzed the prevalence of three common *VDR* polymorphisms (*TaqI-rs731236*, *Bsml-rs1544410*, and *ApaI-rs7975232*) and investigated their potential relationships with the quality of life in a well-characterized cohort of Polish patients with PBC and AIH.

Calcium and vitamin-D supplementation (400–800 IU/day) is recommended to both patients with AIH, who are frequently on long-term steroids, and with PBC, who in their majority are postmenopausal females prone to osteoporosis and impaired vitamin-D absorption secondary to cholestasis. Vitamin D, operating through a nuclear receptor (VDR), is an important modulator of immune processes that adjust both types of immune response [27] by strengthening innate immunity and suppressing acquired immunity reactions [9].

Interestingly, in our study, in the two examined autoimmune liver diseases, both the CC *TaqI* and the AA *ApaI* genotype occurred more frequently than in the controls, and both the C allele of *TaqI* and the A allele of *ApaI* were risk alleles for PBC and AIH. Our results are in contrast to a study in which a significant association of the *TaqI* but not the *ApaI* polymorphism within German AIH patients was reported [28]. However, results from the study that included both patients with AIH and patients with PBC in one merged group showed an increased incidence of the A allele of *ApaI* [29]. These findings may be explained by the genetic heterogeneity that exists in different populations. For instance, the distribution of *BsmI*, *ApaI*, and *TaqI* gene variants was reported to be dissimilar in healthy Chinese controls as compared to healthy Caucasian controls [30]. In our patients with AIH, the A allele and AA genotype of the *BsmI* variant were more prevalent compared to the controls. These results are in a line with reports showing the link between *VDR* polymorphisms and autoimmunity. Thus, the *TaqI*, *BsmI*, *and ApaI* polymorphisms of *VDR* gene are the most widely reported as being closely linked with a high risk of autoimmune diseases including PBC [31], multiple sclerosis (*BsmI* AA) [32], Type 1 diabetes (*TaqI* T [33] or *BsmI* AA [34]), and systemic lupus erythematosus (*BsmI* AA) [35,36]. The functional consequence of these *VDR* polymorphisms is important in determining the potential effect on inflammatory mediators in autoimmune diseases. Vitamin D stimulates the development of Th2 cells and the production of anti-inflammatory interleukins. Therefore, reduced signal transduction due to polymorphic variants of the *VDR* gene might skew the immune response to the Th1 pathway that was implicated in the progress of organ-specific autoimmune diseases. *ApaI* and the *BsmI* polymorphisms do not change the amino acid sequence of the VDR protein but may affect gene expression through the alteration of mRNA stability (the disruption of splice sites for mRNA transcription or a change in intronic regulatory elements) [28], and it was demonstrated that the ApaI variant was positively associated with the serum concentration of 25 (OH)_2_D_3_ [37]. Moreover, IFN gamma production upon anti-CD3 stimulation in the AA [BB] genotype of *BsmI* was significantly higher than that in the AG (Bb) and GG (bb) genotype groups, which showed that the polyclonal T-cell response in BB genotype patients was Th1-dominant [34]. In turn, the *TaqI* polymorphism is involved in the regulation of the stability of VDR mRNA, and the TT genotype modulates VDR expression and confers protection against multiple sclerosis [17,28,38].

Our previous report, on a smaller group of PBC patients (n = 143), showed that there is an association between the *TaqI* and *BsmI*, a predisposition to earlier onset of liver damage and a more severe manifestation of disease [39]. In this study on a larger group of PBC patients (n = 230), it was confirmed that the *TaqI CC* and *BsmI GG* genotypes are related to the degree of liver morphologic damage, as assessed by severity of liver fibrosis (Stage IV on histology). On the basis of the results of this study, we validated our previous conclusion that these variants of the *VDR* gene may prompt more severe liver injury and a worse course of primary biliary cholangitis. However, the interpretation of the role of *TaqI and BsmI* variants in the development of liver fibrosis is hindered because only limited information is available on the functional changes induced by these variants of the *VDR* gene.

In general, little is known about the impact of *VDR* polymorphisms on the quality of patients’ life since previous studies mostly focused on the prevalence of each polymorphism in autoimmune diseases but not on their relation with the clinical course of the disease [29]. Most studies on the quality of life in patients with PBC or AIH addressed relations between specific aspects of disease, such as fatigue, pruritus, depression, and quality of life after liver transplantation [7,40,41,42,43,44,45,46,47,48,49] but not the impact of *VDR* gene polymorphisms.

In this present study, we showed that there is an association between the *TaqI* variant of the *VDR* gene and impaired well-being of patients with PBC, as measured with general and disease-specific questionnaires. The CC of *TaqI* was associated with worse health-related quality of life, as measured by the generic SF-36. This was mainly due to the decrease in subscores of energy and emotional reactions, both associated with fatigue and significant cognitive impairment. Moreover, our analysis of the PBC-40 domains showed that PBC patients’ quality of life was significantly impaired in the carriers of CC and CT genotype of *TaqI*, and fatigue and cognitive function were the most affected domains. In contrast to patients with PBC, among AIH patients, all three variants of *VDR* polymorphism, i.e., *TaqI*, *BsmI*, and *ApaI*, affected health-related quality of life. We observed that the CC and CT of *TaqI* were associated with worse physical and social functioning, while the AA genotype of *BsmI* had physical problems and worse overall health. Interestingly, *ApaI* was the polymorphic variant that mostly affected the quality of life of AIH patients. Our study clearly indicated that the AA homozygotes of *ApaI* variant had disturbed or maladaptive emotional responses and mental disorders, while the CC homozygotes scored fewer points for social functioning. We observed a similar phenomenon in patients with another liver disease of presumed autoimmune background, namely, primary sclerosing cholangitis (PSC) [19]. In that group of patients, the SF-36 questionnaire showed that the C allele of *ApaI* was associated with a reduction of physical, emotional, and mental function and worse overall health. Furthermore, the PBC-40 and PBC-27 questionnaires confirmed that the C allele was associated with itching, fatigue, and the impairment of cognitive functions. Correspondingly, individuals who were AA homozygotes (noncarriers of the C allele of *ApaI*) had higher summary scores for the physical and mental disorders measured with SF-36; they suffered less from itching or fatigue and did not have significant cognitive impairment [19]. The observed association between *VDR* polymorphisms and quality of life is of importance to daily clinical practice because patients with AIH struggle with serious symptoms that significantly affect their well-being, including mood disturbance, cognitive dysfunction, chronic fatigue, decreased physical activity, and a high rate of previously unrecognized severe symptoms of depression and anxiety [7,50]; thus, the presence of the CC *ApaI* variant may result in those symptoms worsening.

Perhaps the major limitation of this study, related to its retrospective nature, is the lack of data on Vitamin D serum levels in analyzed patients and thus the inability to correlate these levels with analyzed polymorphisms. This problem has also been noted in other, similar studies. Serum levels of Vitamin D depend not only on whether patient supplements it but also on patient’s diet and several other factors. Therefore, normal serum levels of Vitamin D could be related to its regular intake, and this would certainly be independent of the presence of VDR polymorphisms. Judgement based on information provided by the patient regarding Vitamin D/calcium supplementation can also be inaccurate in view of widely reported non-adherence to drugs, which do not directly relieve symptoms such as Vitamin D deficiency or hypertension. Thus, in a real-world situation, it is very difficult to reliably assess relationship between real serum Vitamin D levels and VDR polymorphisms. For the same reason, we were not able to study a direct effect of serum Vitamin D levels on patients QoL.

## 5. Conclusions

We observed a significant dominance of the CC *TaqI* and AA *ApaI* genotypes in patients with PBC and AIH. Moreover, the impaired quality of life in patients with AIH was significantly associated with the presence of the AA *ApaI* variant of the *VDR* gene. Awareness of this association can contribute to a deeper understanding of the mechanisms responsible for the occurrence of symptoms associated with poorer quality of life, thereby offering the chance to improve the care of AIH patients.

## Figures and Tables

**Table 1:** Demographic data of analyzed subjects.

Feature	PBC (*n* = 230)	AIH (*n* = 142)	Controls (*n* = 376)
Age (years)	55 (28–90)	32 (24–64)	28 (18–66)
Gender (F/M)	213/17	111/31	344/32
ALT (IU/L) (normal: 5–35)	47.0 (10.0–987.0)	113.8 (1.0–1542.0)	W.N.R.
ALP (IU/L) (normal: 40–120)	286.0 (37.0–1344.0)	100.1 (23.0–344.0)	W.N.R.
GGT, IU/L (normal: 5–35)	177.0 (11.0–1932.0)	102.9 (8.0–766.0)	W.N.R.
Bilirubin (mg/dL) (normal: 0.2–1.0)	0.9 (0.2–45.0)	1.6 (0.2–34.1)	N.D.
Albumin (g/dL) (normal: 3.5–4.5)	4.0 (2.1–5.8)	4.0 (2.0–5.0)	N.D.
Cholesterol (mg/dL) (normal: <190)	217.0 (50.0–1096.0)	182.0 (53.0–319.0)	N.D.
TG (mg/dL) (normal: <150)	105.0 (47.0–681.0)	91.0 (27.0–252.0)	N.D.

PBC: primary biliary cholangitis; AIH: autoimmune; ALT: alanine aminotransferase; ALP: alkaline phosphatase; GGT: gamma-glutamyl transferase; TG: triglycerides; W.N.R.: within normal range; N.D. not done.

**Table 2:** Genotype counts for vitamin-D receptor (*VDR*) polymorphisms (rs731236, rs1544410, rs7975232) in PBC, AIH, and control subjects.

Frequencies	Controls (%) *n* = 376	PBC (%) *n* = 230	*P* * PBC vs. Control	X^2^	OR (95% CI)	AIH (%) *n* = 142	*P* * AIH vs. Control	X^2^	OR (95% CI)
**Taql (rs731236)**									
TT (TT)	172 (45.7%)	28 (12.2%)	**<0.001**	72.7	0.2 (0.1–0.3)	48 (33.8%)	**0.01**	6.0	0.6 (0.4–0.9)
CT (tT)	160 (42.6%)	118 (51.3%)	**0.04**	4.4	1.4 (1.0–2.0)	62 (43.7%)	0.8	0.05	1.0 (0.7–1.5)
CC (tt)	44 (11.7%)	84 (36.5%)	**<0.001**	52.8	4.3 (2.9–6.6)	32 (22.5%)	**0.002**	9.7	2.2 (1.3–3.6)
Bsml (rs1544410)									
AA (BB)	52 (13.8%)	25 (10.9%)	0.3	1.1	0.8 (0.5–1.3)	34 (23.9%)	**0.006**	7.6	2.0 (1.2–3.2)
GA (bB)	173 (46%)	109 (47.4%)	0.7	0.1	1.1 (0.8–1.5)	60 (42.3%)	0.4	0.6	0.9 (0.6–1.3)
GG (bb)	151 (40.2%)	96 (41.7%)	0.7	0.1	1.1 (0.8–1.5)	48 (33.8%)	0.2	1.76	0.8 (0.5–1.1)
Apal (rs7975232)									
AA (AA)	74 (19.7%)	63 (27.4%)	**0.03**	4.8	1.5 (1.0–2.3)	46 (32.4%)	**0.002**	9.4	2.0 (1.3–3.0)
CA (aA)	196 (52.1%)	111 (48.2%)	0.3	0.8	0.9 (0.6–1.2)	61 (43.0%)	0.06	3.5	0.7 (0.5–1.0)
CC (aa)	106 (28.2%)	56 (24.4%)	0.3	1.0	0.8 (0.6–1.2)	35 (24.6%)	0.4	0.6	0.8 (0.5–1.3)

* Chi-squared test of association (Pearson); PBC: primary biliary cholangitis; AIH: autoimmune hepatitis; OR: odds ratio; CI: confidence interval. Bold font indicates statistical significance.

**Table 3:** Allele association for VDR in PBC, AIH, and control subjects.

SNP	Allele	Controls *n* = 376 (%)	PBC *n* = 230 (%)	*P* * PBC vs. Control	X^2^	OR (95%CI)	AIH *n* = 142 (%)	*P* * AIH vs. Control	X^2^	OR (95%CI)
TaqI rs731236	T/C (T/t)	504/248 (67.0/33.0)	174/286 (38.0/62.0)	**<0.001**	98.7	3.3 (2.6–4.3)	158/126 (55.6/44.4)	**<0.001**	11.6	1.6 (1.3–2.1)
BsmI rs1544410	A/G (B/b)	277/475 (36.8/63.2)	159/301 (34.6/65.4)	0.4	0.6	1.1 (0.9–1.4)	128/156 (45.0/55.0)	**0.02**	5.9	1.4 (1.1–1.9)
ApaI rs7975232	A/C (A/a)	344/408 (45.7/54.3)	237/223 (51.4/48.6)	**0.05**	3.8	0.8 (0.6–1.0)	153/131 (54.0/46.0)	**0.02**	5.4	1.4 (1.1–1.8)

* Chi-squared test of association (Pearson); PSC: primary biliary cholangitis; AIH: Autoimmune hepatitis; OR: odds ratio; CI: confidence interval. Bold font indicates statistical significance.

**Table 4:** Relationship between *TaqI* polymorphism and features of quality-of-life scales in PBC group.

Domain	*TaqI (rs731236 T/C)*					
	CC (tt)	CT (tT)	TT (TT)	*P* * CC vs CT	*P* * CC vs TT	*P* * TT vs CT
**SF-36**						
Physical Functioning	57.0 ± 3.2	59.5 ± 2.3	67.0 ± 5.3	0.5	0.09	0.2
Role-Physical	36.5 ± 4.3	34.0 ± 3.7	50.0 ± 8.3	0.6	0.1	0.05
Bodily Pain	54.4 ± 3.2	55.4 ± 2.4	61.0 ± 5.8	0.8	0.3	0.3
General Health	43.4 ± 1.9	43.6 ± 1.7	45.1 ± 3.4	0.9	0.7	0.7
Vitality	44.5 ± 2.4	47.5 ± 1.9	57.0 ± 4.8	0.3	**0.01**	**0.04**
Social Functioning	59.2 ± 3.0	61.2 ± 2.3	69.0 ± 5.3	0.6	0.08	0.1
Role-Emotional	47.4 ± 5.0	49.7 ± 4.2	69.0 ± 7.8	0.7	**0.03**	**0.04**
Mental Health	59.0 ± 2.0	59.9 ± 1.9	67.2 ± 4.3	0.7	0.06	0.09
Physical Component Summary	47.8 ± 2.6	47.8 ± 2.0	55.0 ± 4.7	0.8	0.1	0.1
Mental Component Summary	52.2 ± 2.5	54.4 ± 2.1	65.0 ± 5.0	0.5	0.2	0.4
**PBC-40**						
Other Symptom	17.0 ± 0.6	17.3 ± 0.5	16.1 ± 1.0	0.6	0.4	0.2
Itch	6.0 ± 0.5	5.4 ± 0.4	5.0 ± 0.9	0.4	0.3	0.6
Fatigue	29.6 ± 1.2	30.4 ± 1.0	26.1 ± 1.8	0.6	0.1	**0.04**
Cognitive function	14.2 ± 0.7	14.1 ± 0.5	11.7 ± 1.0	0.8	**0.04**	**0.04**
Social and Emotional function	31.5 ± 10.7	31.6 ±11.5	28.0 ±11.0	0.9	0.1	0.08

* ANOVA with Fisher’s protected least significant difference (PLSD). Letters enclosed in brackets represent previously described nomenclature derived from restriction-fragment length polymorphism (RFLP) analysis. Bold font indicates statistical significance.

**Table 5:** Relationship between *TaqI* polymorphism and features of quality-of-life scales in AIH group.

Domain	*TaqI (rs731236 T/C)*					
	CC (tt)	CT (tT)	TT (TT)	*P* * CC vs *CT*	*P* * CC vs *TT*	*P* * TT vs *CT*
**SF-36**						
Physical Functioning	73.0 ± 4.6	79.0 ± 2.8	73.6 ± 3.4	0.2	0.9	0.2
Role-Physical	47.7 ± 7.6	66.1 ± 5.5	55.7 ± 5.8	**0.04**	0.4	0.2
Bodily Pain	67.4 ± 5.2	74.2 ± 3.1	69.4 ± 4.1	0.3	0.7	0.3
General Health	47.5 ± 3.5	49.9 ± 2.5	47.5 ± 3.5	0.6	0.9	0.6
Vitality	53.3 ± 3.5	54.3 ± 2.5	51.6 ± 2.8	0.8	0.7	0.5
Social Functioning	67.2 ± 4.1	75.4 ± 3.2	65.1 ± 3.7	0.1	0.7	**0.03**
Role-Emotional	59.4 ± 7.3	74.7 ± 4.8	68.1 ± 5.8	0.08	0.3	0.4
Mental Health	63.5 ± 2.9	66.3 ± 2.6	61.3 ± 2.5	0.5	0.6	0.2
Physical Component Summary	58.9 ± 4.2	67.3 ± 2.7	61.6 ± 3.5	0.09	0.6	0.2
Mental Component Summary	60.8 ± 3.8	67.7 ± 2.8	61.5 ± 3.0	0.1	0.9	0.1
**STAI**						
STAI1	47.4 ± 0.8	45.9 ± 0.8	46.2 ± 0.8	0.2	0.4	0.8
STAI2	45.1 ± 0.9	45.3 ± 0.7	46.0 ± 0.8	0.9	0.5	0.5
**MFIS**						
Physical	13.7 ± 1.6	14.1 ± 1.0	14.1 ± 1.1	0.8	0.9	0.9
Cognitive	13.2 ± 1.6	11.7 ± 0.9	12.2 ± 1.0	0.4	0.6	0.7
Psychosocial	2.8 ± 0.4	2.5 ± 0.2	3.0 ± 0.3	0.4	0.7	0.2
MFIS Score	13.7 ± 1.6	14.1 ± 1.0	14.1 ± 1.1	0.8	0.9	0.9
**PHQ-9**						
PHQ-9	7.2 ± 0.8	6.0 ± 0.6	7.7 ± 0.8	0.3	0.6	0.08

* ANOVA with Fisher’s protected least significant difference (PLSD); The letters enclosed in square brackets represent previously described nomenclature derived from a restriction-fragment length polymorphism (RFLP) analysis. Bold font indicates statistical significance.

**Table 6:** Relationship between *BsmI* polymorphism and features of quality-of-life scales in AIH group.

Domain	*BsmI (rs1544410 A/G)*					
	GG (bb)	GA (bB)	AA (BB)	*P* * GG vs GA	*P* * GG vs AA	*P* * AA vs GA
**SF-36**						
Physical Functioning	73.9 ± 3.4	80.5 ± 2.5	72.3 ± 4.9	0.1	0.8	0.1
Role-Physical	56.2 ± 5.7	66.7 ± 5.4	49.3 ± 7.6	0.2	0.5	0.05
Bodily Pain	70.3 ± 4.1	73.5 ± 2.9	65.1 ± 5.2	0.5	0.4	0.1
General Health	48.9 ± 3.4	51.0 ± 2.5	46.2 ± 3.7	0.6	0.6	0.3
Vitality	53.3 ± 2.6	54.5 ± 2.4	52.0 ± 3.7	0.7	0.8	0.5
Social Functioning	65.8 ± 3.7	75.2 ± 3.0	67.0 ± 4.2	0.05	0.8	0.1
Role-Emotional	68.7 ± 5.9	75.5 ± 4.7	60.8 ± 7.2	0.4	0.4	0.08
Mental Health	63.5 ± 2.4	67.1 ± 2.4	61.9 ± 3.2	0.3	0.7	0.2
Physical Component Summary	62.3 ± 3.3	67.9 ± 2.5	58.2 ± 4.4	0.2	0.4	**0.04**
Mental Component Summary	62.9 ± 3.0	68.1 ± 2.6	60.4 ± 4.0	0.2	0.6	0.09
**STAI**						
STAI1	47.1 ± 0.8	45.6 ± 0.8	46.9 ± 0.8	0.1	0.8	0.3
STAI2	46.2 ± 0.8	45.3 ± 0.8	44.7 ± 0.8	0.4	0.2	0.6
**MFIS**						
Physical	14.0 ± 1.0	14.1 ± 1.0	14.2 ± 1.6	0.9	0.9	0.9
Cognitive	12.4 ± 1.0	11.4 ± 0.8	13.0 ± 1.5	0.5	0.7	0.3
Psychosocial	3.0 ± 0.3	2.6 ± 0.2	2.9 ± 0.4	0.4	0.8	0.6
MFIS Score	29.4 ± 2.0	28.2 ± 1.9	30.1 ± 3.3	0.7	0.9	0.6
**PHQ-9**						
PHQ-9	7.0 ± 0.8	6.2 ± 0.6	7.4 ± 0.8	0.4	0.7	0.2

* ANOVA with Fisher’s protected least-significant difference (PLSD). Letters enclosed in brackets represent previously described nomenclature derived from restriction-fragment length polymorphism (RFLP) analysis. Bold font indicates statistical significance.

**Table 7:** Relationship between *ApaI* polymorphism and features of quality-of-life scales in AIH group.

Domain	*ApaI (rs7975232 C/A)*					
	CC (aa)	CA (aA)	AA (AA)	*P* * CC vs CA	*P* * CC vs AA	*P* * AA vs CA
**SF-36**						
Physical Functioning	73.6 ± 4.0	80.5 ± 2.3	74.0 ± 3.9	*0.1*	*0.9*	*0.1*
Role-Physical	56.1 ± 6.9	68.1 ± 5.0	49.5 ± 6.3	*0.2*	*0.5*	***0.02***
Bodily Pain	71.7 ± 4.9	72.7 ± 3.1	67.8 ± 4.1	*0.8*	*0.5*	*0.3*
General Health	47.3 ± 3.8	51.5 ± 2.5	47.5 ± 3.1	*0.3*	*0.9*	*0.3*
Vitality	52.2 ± 3.1	56.5 ± 2.3	50.4 ± 2.8	*0.3*	*0.7*	*0.09*
Social Functioning	63.8 ± 3.9	76.4 ± 2.8	66.6 ± 3.9	***0.01***	*0.6*	***0.04***
Role-Emotional	64.7 ± 6.9	80.6 ± 4.2	58.5 ± 6.1	*0.05*	*0.4*	***0.003***
Mental Health	62.5 ± 2.7	67.8 ± 2.2	60.9 ± 2.9	*0.2*	*0.7*	***0.04***
Physical Component Summary	62.5 ± 2.7	67.9 ± 2.2	60.9 ± 2.9	*0.2*	*0.7*	***0.04***
Mental Component Summary	62.2 ± 3.9	68.2 ± 2.5	58.7 ± 3.5	*0.2*	*0.6*	***0.04***
**STAI**						
STAI1	46.5 ± 1.0	45.7 ± 0.7	46.9 ± 0.8	*0.5*	*0.7*	*0.2*
STAI2	46.3 ± 0.9	45.2 ± 0.6	45.0 ± 0.8	*0.4*	*0.3*	*0.8*
**MFIS**						
Physical	13.2 ± 1.2	14.8 ± 1.0	13.9 ± 1.2	*0.3*	*0.7*	*0.6*
Cognitive	11.5 ± 1.1	12.6 ± 0.9	12.3 ± 1.2	*0.5*	*0.3*	*0.8*
Psychosocial	2.8 ± 0.3	3.0 ± 0.2	2.6 ± 0.3	*0.6*	*0.7*	*0.3*
MFIS Score	27.4 ± 2.4	30.4 ± 1.9	28.8 ± 2.6	*0.4*	*0.8*	*0.6*
**PHQ-9**						
PHQ-9	7.1 ± 0.9	6.6 ± 0.6	6.9 ± 0.7	*0.7*	*0.8*	*0.8*

* ANOVA with Fisher’s protected least-significant difference (PLSD). Letters enclosed in brackets represent previously described nomenclature derived from restriction-fragment length polymorphism (RFLP) analysis. Bold font indicates statistical significance.
